# *Campylobacter* in Wild Birds: Is It an Animal and Public Health Concern?

**DOI:** 10.3389/fmicb.2021.812591

**Published:** 2022-02-10

**Authors:** Nejash A. Ahmed, Timur Gulhan

**Affiliations:** Department of Veterinary Microbiology, Faculty of Veterinary Medicine, Ondokuz Mayis University, Samsun, Turkey

**Keywords:** *Campylobacter*, public health, wild birds, animal health, foodborne infections

## Abstract

Campylobacteriosis continues to be one of the leading causes of foodborne bacterial zoonotic infections worldwide. Despite its public health importance, the status of this disease in wild birds and the possibility of transmission from wild birds to domestic animals and humans have not been clearly elucidated yet. This article reviews the available literature with the aim of making a comprehensive manuscript on this disease status in wild birds and the possibility of interspecies transmission. *Campylobacter* has been isolated from various species of wild birds worldwide, with *C. jejuni* being the most commonly isolated species. The prevalence of *Campylobacter* in wild birds may vary depending on several factors like geographical location, season, the bird’s health status, bird species, sample type, the method used, and ecological factors. Molecular studies over the past two to three decades have characterized *Campylobacter* strains isolated from wild birds and have come up with results that fall into two categories. The first are those that report overlapping strains among human, domestic animal, and wild bird isolates. The results of the studies under this category emphasize that wild birds carry strains of *Campylobacter*, which are indistinguishable from domestic animals and humans and are therefore an important public and animal health concern. In contrast, the studies under the second category highlight significant differences in *Campylobacter* population structure among these hosts. Despite the controversiality and the inadequacy of current research to draw a full conclusion, the role of wild birds in the epidemiology of *Campylobacter* should not be undermined as drug-resistant strains, especially resistance to tetracycline and fluoroquinolones, are increasingly documented. In addition, source attribution studies have linked human cases of *Campylobacter* infections to wild birds. Therefore, the role of wild birds in the epidemiology of *Campylobacter* infection should not be neglected. However, in order to determine disease status in wild birds and the precise role of wild birds in domestic animals and human health, detail-oriented epidemiological investigations characterizing the genetic relatedness of isolates from the respective species and environment through one health approach are warranted.

## Introduction

Campylobacteriosis is currently considered to be the most commonly reported zoonotic bacterial foodborne gastroenteritis worldwide (Silva et al., 2011; Igwaran and Okoh, 2019). Over the past decades, a rise in its incidence has been evidenced in different parts of the world, including both developed and developing countries (Kaakoush et al., 2015; Igwaran and Okoh, 2019). The World Health Organization (WHO) estimated that at least 96 million cases of enteric infections worldwide are associated with *Campylobacter* species annually (Havelaar et al., 2015). According to the “European Union One Health 2019 zoonoses report,” campylobacteriosis ranked first as the most commonly reported zoonoses in European Union member countries, with 220,682 confirmed human cases in 2019 alone (European Food Safety Authority [EFSA], and European Centre for Disease Prevention and Control [ECDC], 2021). Therefore, campylobacteriosis is a disease of public health concern globally (Igwaran and Okoh, 2019).

Although various animal species, including wild birds, are known sources of *Campylobacter* infection (Zenebe et al., 2020; Mughini-Gras et al., 2021), poultry is accepted to act as reservoirs of 50–80% of *Campylobacter* infections in humans, and cattle are considered to act as reservoirs of 20–30% of human infections (European Food Safety Authority [EFSA], 2010). One study conducted in the Baltic States showed that clinical cases of *Campylobacter jejuni* (*C. jejuni*) infections in humans were associated with sources from poultry (88.3%), cattle (9.4%), and wild birds (2.3%) (Maësaar et al., 2020). Another study that used multilocus sequence typing (MLST) to determine the infection source linked 64.5% of human *C. jejuni* infection to poultry, with cattle and wild birds accounting for 25.8 and 2.3%, respectively (Levesque et al., 2013). Thus, poultry and cattle are generally accepted as significant sources of human campylobacter infection (Mughini-Gras et al., 2021). Even though wild bird *Campylobacter* carriage is much lower than poultry (Maësaar et al., 2020; Zhang and Sahin, 2020), wild birds are known to act as significant reservoirs, implying that they may have a role in spreading the bacteria to the environment (Navarro-Gonzalez et al., 2016; Aksomaitiene et al., 2019; Marotta et al., 2019; Maësaar et al., 2020).

Currently, *Campylobacter* has been isolated from several species of wild birds (e.g., birds of prey, waterfowl, crows, pigeons, gulls, and others) in different areas in the world, including America, Australia, Asia, Europe, and Africa (Keller et al., 2011; Konicek et al., 2016; Moré et al., 2017; Du et al., 2019; Antilles et al., 2021; Kürekci et al., 2021). In addition to pathogen detection, antibiotic resistance, one of the global challenges of the current century (Sabtu et al., 2015), is also being reported in *Campylobacter* spp. isolated from wild birds. In particular, resistance to tetracycline and fluoroquinolones is increasingly documented (Jurado-Tarifa et al., 2016; Indykiewicz et al., 2021; Mencía-Gutiérrez et al., 2021; Russo et al., 2021).

Due to their significant reservoir role, *Campylobacter* status in poultry and cattle has been extensively studied, demonstrating the significant attention paid to the risk of acquiring *Campylobacter* infection from food sources (Mohan, 2015). However, only limited information is available regarding the role of wild birds as a reservoir of pathogens like *Campylobacter*, which can be related to the difficulty in collecting samples from wild birds (Antilles et al., 2015; Mencía-Gutiérrez et al., 2021). Despite the scarcity of research, the available literature indicates that contamination of equipment and surfaces with wild bird feces can be a risk for human health (French et al., 2009). Wild birds are shown to be one of the leading causes of contamination of surface water (Mulder et al., 2020), and source tracking studies have linked human cases of *Campylobacter* infections to wild birds (Gardner et al., 2011). For example, a molecular study conducted in the United Kingdom related 476–543 annual clinical cases of human *Campylobacter* infection to wild birds, emphasizing the importance of wild birds in human campylobacteriosis (Cody et al., 2015). Another molecular study from the United States also linked the human outbreak of *Campylobacter* infection due to raw peas consumption to wild birds (Kwan et al., 2014).

Despite the public and economic importance of *Campylobacter* infections, the status of this disease in wild birds and the likelihood of transmission from wild birds to domestic animals and humans have not been clearly determined yet. However, understanding the status of this disease in wild birds and the possibility of interspecies transmission is necessary to designing applicable policies. To date, no article has exclusively reviewed the status of *Campylobacter* in wild birds and its associated public and animal health significance, except a few articles (Abulreesh et al., 2006; Dhama et al., 2008; Benskin et al., 2009; Whiley et al., 2013; Clark, 2014; Navarro-Gonzalez et al., 2016; Elmberg et al., 2017; Smith et al., 2020) reviewing pathogens in general or in a specific host. Therefore, this article aims to review the available literature on *Campylobacter* in wild birds and summarize the current understanding of interspecies transmission to show what is currently known about its public and animal health importance.

## Historical and General Information on *Campylobacter*

According to available information, Theodor Escherich was thought to have made the first report on *Campylobacter* in 1886 (Silva et al., 2011). Despite this, *Campylobacter* was not recognized as a primary disease-causing agent in humans until the 1970s (Butzler, 2004), which is thought to be due to difficulties in culturing and identifying it (Sheppard and Maiden, 2015). In the case of livestock in general, the diseases due to *Campylobacter* have been well documented since the beginning of the twentieth century (Butzler, 2004; Hlashwayo et al., 2020). The role of wild birds as carriers of *Campylobacter* has also long been recognized (Luechtefeld et al., 1980; Kapperud and Rosef, 1983; Kinjo et al., 1983; Fukuyama et al., 1986). Even though it is unclear whether it was the first report, Luechtefeld et al. (1980) reported *C. jejuni* carriage in migratory waterfowl in samples collected from 1978 to 1980 in Northern Colorado, United States. Following this, several researchers reported *Campylobacter* carriage in various wild bird species such as pigeons, gulls, crows, starlings, and others (Kinjo et al., 1983; Fukuyama et al., 1986; Ito et al., 1988; Whelan et al., 1988; Fernández et al., 1996).

The currently used *Campylobacter* genus name (previously known as *Vibrio* spp.) was proposed by Sébald and Véron for the first time in 1963 (Frasao et al., 2017). The causative agent of campylobacteriosis includes various pathogenic species of *Campylobacter*, which are small (0.2–0.8 μm × 0.5–5 μm), micro-aerophilic, and spiral Gram-negative bacteria belonging to the family *Campylobacteriaceae*, class Epsilonproteobacteria, and phylum Proteobacteria (Silva et al., 2011; Muralidharan et al., 2016). Currently, about 53 *Campylobacter* species and 16 subspecies have been documented (accessed November 20, 2021), (LPSN), including those considered pathogenic to humans and livestock (Humphrey et al., 2007). Among these species, thermophilic *Campylobacter* (for example, *C. jejuni* and *C. coli*) are essential zoonotic pathogens that cause gastroenteritis in humans worldwide (Kreling et al., 2020). The rise in the number of species associated with animal and human infections is believed to be why this bacterium needs significant attention (Igwaran and Okoh, 2019). Most *Campylobacter* species (except *C. gracilis*, and *C. showae*) can move using amphitrichous flagella. If two *Campylobacter* cells are found together, they appear in an “S” shape, resembling a flying gull’s wing (Silva et al., 2011), and the name *Campylobacter*, which is taken from the Greek word “campylos,” also describes this “S” shape morphology (Kreling et al., 2020).

## Identification and Characterization of *Campylobacter*

For the identification and characterization of *Campylobacter*, a variety of phenotypic (e.g., culture) and genotypic methods [e.g., MLST, polymerase chain reaction (PCR)], each with its own pros and cons, have been documented (Eberle and Kiess, 2012). Bacterial culturing and biochemical tests have long been used to characterize pathogens (Ferone et al., 2020). The commonly performed detection method of *Campylobacter* spp. from avian fecal samples relies on culturing techniques, including directly plating cecal/fecal materials onto selective agar plates or pre-enrichment in “selective enrichment broth” followed by “selective plating.” However, the success of the bacteria recovery rate in the former method depends on the bacteria’s number present, and hence the method may not show satisfactory recovery from animal and avian feces (Abulreesh et al., 2006). Even though pre-enrichment may not always result in a higher recovery rate than direct plating (Zhang and Sahin, 2020), in the case of a low number of bacteria, recovery rates may be improved if pre-enrichment is performed and followed by selective plating (Abulreesh et al., 2006). Since this bacterium requires special conditions (fastidious growth requirements) such as low oxygen concentrations and enriched media, traditional culture-based identification methods are challenging, and species differentiation is also difficult as there are limited biochemical tests (Al Amri et al., 2007; Frasao et al., 2017).

Currently, molecular-based methods such as PCR, MLST, and pulsed-field gel electrophoresis (PFGE) are frequently being used for species identification and genotypic characterization of *Campylobacter* isolates obtained from wild birds (Keller and Shriver, 2014; Mohan, 2015; Indykiewicz et al., 2021; Kürekci et al., 2021; Mencía-Gutiérrez et al., 2021). Multiplex PCR, a rapid and accurate method that detects multiple-gene presence/absence in a single reaction mixture, is among the most commonly used methods for differentiating different species of *Campylobacter* (Frasao et al., 2017; Ricke et al., 2019). Several researchers have used this method to identify the species of *Campylobacter* isolates obtained from wild birds (Keller et al., 2011; Ramonaitë et al., 2015; Kürekci et al., 2021). Therefore, mPCR should be considered when the objective is to identify or differentiate different species of *Campylobacter* (Frasao et al., 2017).

“Matrix-assisted laser desorption ionization time-of-flight mass spectrometry (MALDI-TOF MS)” is another method employed to differentiate *Campylobacter* at the genus and/or species level (Bessè De et al., 2011; Dudzic et al., 2016). Compared with the morphology-based conventional methods, this method is fast, cost-effective, and more reliable for identifying *Campylobacter* spp. (Bessè De et al., 2011). Recently, researchers have pointed out the usage of MALDI-TOF MS in the differentiation of *Campylobacter* isolates obtained from wild birds (Dudzic et al., 2016; Lawton et al., 2018). For example, a more recent study by Kürekci et al. (2021) identified *Campylobacter* isolates of wild bird origin to genus level using MALDI-TOF MS. The study by Dudzic et al. (2016), who used culture-based detection, MALDI-TOF, and PCR, reported that 35 *Campylobacter* sp. isolates obtained from pigeons were all confirmed to be *Campylobacter* at the genus level by MALDI-TOF and contained 16 rRNA genes specific for *Campylobacter* spp. by PCR. Lawton et al. (2018) comparatively investigated *Campylobacter* isolates using MALDI-TOF MS, PCR, and WGS and reported 100% agreement in species identification among these methods.

MLST is the sequence-based molecular typing method used for evaluating strain relationships. Although it has a low resolution compared to WGS, MLST can provide highly discriminatory pathogen clustering results by examining the sequences of 7 housekeeping genes (Tong et al., 2021). This technique has been used in assessing *Campylobacter* strain overlap among different species of wild birds, poultry, and humans (Colles et al., 2008; French et al., 2009; Hughes et al., 2009; Du et al., 2019; Wei et al., 2019; Jurinoviæ et al., 2020). Since the application of MLST is restricted to the sequence type characterization, whole-genome or core-genome MLST techniques are preferred for the accurate differentiation of related strains (Marotta et al., 2020). Currently, technological advancements have enabled the use of a more advanced WGS technique, which reveals an organism’s entire DNA make-up (Franz et al., 2016). WGS provides higher-resolution phylogenetic information than other methods, such as MLST, making it ideal for source attribution studies and comparing strains from different origins (Tong et al., 2021).

## Epidemiology of *Campylobacter* in Wild Birds

Wild birds are a well-known significant natural reservoir of *Campylobacter* species (especially thermophilic *Campylobacter* spp., i.e., *C. jejuni*, *C. coli*, and *C. lari*) (Krawiec et al., 2017; Mencía-Gutiérrez et al., 2021). This bacterium has been isolated from several species of wild birds (birds of prey, waterfowl, crows, pigeons, gulls, geese, and others) in different areas in the world, including Africa, America, Europe, Australia, and Asia, showing its global distribution (Keller et al., 2011; Konicek et al., 2016; Moré et al., 2017; Vogt et al., 2018; Du et al., 2019; Jurinoviæ et al., 2020; Antilles et al., 2021; Kürekci et al., 2021).

Transmission in wild birds is considered to be through fecal–oral, when the birds are foraging near domestic animals, which may result in the spread of pathogen over long distances (Taff and Townsend, 2017). Although avian species, including domestic poultry (Griekspoor et al., 2013) and wild birds (Smith et al., 2020), are known to be asymptomatic carriers of *Campylobacter*, lower survival rates and/or poor body condition have been encountered in infected birds when compared with healthy birds (Waldenström et al., 2010; Taff and Townsend, 2017). For example, in the study conducted by Taff and Townsend (2017), who compared crows infected with *C. jejuni* with uninfected crows with the aim of assessing its impact on body condition and survival of crows, the infected crows were found in poor body condition compared to uninfected crows.

Among *Campylobacter* spp., *C. jejuni* is the most frequently detected species in several wild bird species, as demonstrated by several researchers (Dipineto et al., 2014; Weis et al., 2014; Krawiec et al., 2017; Gargiulo et al., 2018; Antilles et al., 2021; Indykiewicz et al., 2021; Mencía-Gutiérrez et al., 2021). For example, in one study conducted in Italy, *C. jejuni* was detected in all isolates (49/49) from birds of prey, and *C. jejuni* and *C. coli* (mixed infections) were detected in 12 isolates with the overall *Campylobacter* sp. prevalence of 33.1% (49/148) (Gargiulo et al., 2018). However, most of the researchers target the thermophilic species due to their public health importance (Table 1), and thus, it seems that attention was not given to other species. A large-scale study by Johansson et al. (2018) assessed the status of *Campylobacter* in various wild birds (covering a total of 2,278 birds) in the remote area of the Antarctic and sub-Antarctic regions and reported different *Campylobacter* spp. like *C. peloridis*, *C. subantarcticus*, and *C. volucris*, in addition to *C. jejuni* and *C. lari*.

**TABLE 1:** Current status and characteristics of *Campylobacter* spp. isolated from various wild birds.

Wild birds	Country/ area	Sample type	Method used	n/N (P%)	Species (%)	Virulence and resistance status	References
Bird of prey	Spain	Cloacal swab	Culture and mPCR	52/689 (7.5)	*C. jejuni* (88.5) *C. coli* (3.8) *C. lari* (3.8)	Resistance to drugs like fluoroquinolones, tetracycline, and streptomycin was detected	Mencía-Gutiérrez et al., 2021
European turtle dove, Eurasian coot, song thrush, quails, and red-crested pochard	Turkey	Cloacal swab	Culture and MALDI-TOF MS + multiplex qPCR + WGS	6/116 (5.2) (song thrushes) 41/44 (93) (Eurasian coots)	*C. jejuni* *C. coli*	The isolates showed susceptibility to tetracycline, (fluoro-) quinolones, gentamicin, and erythromycin, but streptomycin resistance was found.	Kürekci et al., 2021
Black-headed gull (*Chroicocephalus ridibundus*)	Poland	Cloacal swab	Culture and mPCR	35/718 (4.87) (adults) 7/318 (2.22) (chicks)	*C. jejuni* (85.72) *C. coli* (7.14) *C. lari* (7.14)	Resistance to tetracycline (50.00%) and ciprofloxacin (47.62%) was observed	Indykiewicz et al., 2021
Yellow-legged gull and Audouin’s gull	Southern Europe	Cloacal swab	Culture and PCR	93/1,785 (5.2)	*C. jejuni* (94.6) *C. coli* (2.12) *C. lari* (2.12)		Antilles et al., 2021
Yellow-legged gull (*Larus michahellis*)	Italy	Cloacal swab	Culture and mPCR	60/225 (26.7)	*C. coli* 36/60 *C. jejuni* 24/60	More than half of the isolates showed resistance to tetracycline.	Russo et al., 2021
Black-headed gull, yellow-legged gull, Caspian gull, common gull, and herring gull	Croatia	Cloacal swab	Culture and mPCR + MLST	168/643 (26.1)	*C. jejuni* (88.1) *C. lari* (11.3) *C. coli* (0.6)	Resistance to tetracycline fluoroquinolones, gentamicin, and streptomycin was detected	Jurinoviæ et al., 2020
Various	China	Feces sample	Culture and qPCR + MLST	57/520 (10.96)	*C. jejuni*	The *flaA*, *cadF*, and *cdt* genes were identified. 33.3% of the isolates were shown to be MDR	Du et al., 2019
Jackdaws, crows, rooks, magpies	Sweden	Intestinal segment	Culture and, MALDI-TOF MS + sequencing	46/56 (82)	*C. jejuni*		Söderlund et al., 2019
Canada geese (*Branta canadensis*)	Canada	Cloacal samples, fecal swab	Culture and mPCR	48/430 (11.2)	*C. jejuni* *C. coli*		Vogt et al., 2018
Waterbirds, passerines, birds of prey, owls, and other birds	Poland	Feces and cloacal swab	Culture and PCR (for genus) + mPCR	43/700 (6.14)	*C. jejuni* 38/43 (88.37) *C. coli* 5/43 (11.63)	*flaA ceuE*, *cadF*, *cdtA*, *cdtB*, and *cdtC*	Krawiec et al., 2017
Various	India	Fecal sample	Culture and mPCR + sequencing	3/102 (2.94)	*C. jejuni*		Prince Milton et al., 2017
Bird of prey	Italy	Swab (from intestinal mucosa)	Culture and mPCR	49/148 (33.1)	*C. jejuni* (100) *C. coli* (24.48) *		Gargiulo et al., 2018
Various	Austria and Czech Republic	Cloacal swab	Culture and MALDI-TOF MS	149/1,191 (12.5)	*C. jejuni* (88.7) *C. coli* (8.6) *C. lari* (2)		Konicek et al., 2016
Various	South Korea	Cloacal swab/feces sample	Culture and mPCR	332/2,164 (15.3) 213*^a^*	*C. jejuni* 169/213 (79.3) *C. coli* 20/213 (9.3) *C. lari* 1/213 (0.4)	Variable degrees of resistance to antimicrobials was observed	Kwon et al., 2017
Gull (*Larus dominicanus*) and greater crested tern (*Thalasseus bergii*)	South Africa	Cloacal swab	Culture and mPCR + PFGE	32/229 (14)	*C. jejuni* *C. lari*	The isolates showed resistance to tetracycline and quinolones	Moré et al., 2017
Feral pigeons (*Columba livia*)	Canada	Cloacal swab	Culture + biochemical	17/187 (9.1)	*C. jejuni*		Vanessa et al., 2016
Different species of raptors	Spain	Fecal content	Culture and PCR + PFGE	9/387 (2.3)	*C. jejuni* (33.3) *C. coli* (33.3) *C. lari* (11.1)	Resistance to drugs such as tetracycline and ciprofloxacin was detected	Jurado-Tarifa et al., 2016
Crow, pigeon, Eurasian tree sparrow (*Passer montanus*)	Japan	Cloacal swab	Culture and qPCR + sequencing	34/173 (19.7)	*C. jejuni* 32/34 (94.1) *C. coli* 1/34 (2.9) *C. fetus* 1/34 (2.9)	CDT genes, *flaA*, *flaB*, *ciaB*, and *cadF* were found	Shyaka et al., 2015
Crow and pigeon	Lithuania	Feces sample	Culture and mPCR + PCR–RFLP	166/480 (34.6)	*C. jejuni* (100)		Ramonaitë et al., 2015
Waterfowl	Spain	Cloacal swab	Culture and PCR + ERIC-PCR	40/318 (12.5)	*C. coli* (37/40 *C. jejuni* 3/40	All isolates showed susceptibility to quinolones, gentamicin, chloramphenicol, and tetracycline	Antilles et al., 2015
Quail (*Coturnix coturnix*)	Italy	Cloacal swab	Culture and mPCR	15/70 (21.4)	*C. coli* (100) *C. jejuni* (40) *		Dipineto et al., 2014
Various	United States	Cloacal swab and feces sample	Culture and MLST-PCR	72/781 (9.2)	*C. jejuni* *C. coli* *C. lari*		Keller and Shriver, 2014
Pigeon (*Columba livia*)	Italy	Cloacal swab	Culture and mPCR	870/1,800 (48.3)	*C. jejuni* (100)	All isolates carried *cdt* genes.	Gargiulo et al., 2014
Griffon Vultures (*Gyps fulvus*)	Spain	Cloacal swab	Culture + biochemical	1/97 (1.0)	*C. jejuni*		Marin et al., 2014
American crows (*Corvus brachyrhynchos*)	United States	Cloacal/feces swab	Culture and PCR + sequencing	85/127 (66.9)	*C. jejuni* (93) *C. lari*	*cdtA*, *cdtB*, *cdtC*, and *flaA*	Weis et al., 2014
Mallard duck (*Anas platyrhynchos*) and European starlings (*Sturnus vulgaris*)	New Zealand	Fecal sample	Culture and PCR + MLST	539/1,436 (37) 22% *C. jejuni* prevalence	*Campylobacter* spp. (in general) + *C. jejuni*		Mohan et al., 2013
Various	United States	Fecal sample	Culture and mPCR	6/333 (7.2)	*C. jejuni*		Keller et al., 2011
Various	Spain	Fecal sample	Phenotypic and PCR	9/121 (7.4)	*C. jejuni* *C. lari*	Resistance to fluoroquinolones was detected	Molina-Lopez et al., 2011
Waterfowl	Canada	Fecal sample	Culture and direct qPCR	(32) (29)*^b^*	*C. jejuni* (73) *C. coli* (13) *C. lari* (27)		Van Dyke et al., 2010
Various	United Kingdom	Fecal sample	Culture and mPCR + MLST + PFGE	?/2,084 (1.4)	*C. jejuni* *C. coli* *C. lari*		Hughes et al., 2009
Seagull (*Larus* spp.)	Ireland	Fecal sample	Culture + biochemical	28/205 (13.7)	*C. jejuni* *C. lari*		Moore et al., 2002
Black-headed Gulls (*Larus ridibundus*)	Sweden	Fecal sample	Culture and mPCR + PFGE	117/419 (27.9%)	*C. jejuni* *C. coli* *C. lari*		Broman et al., 2002

*N, sample size; n, positive number; P, prevalence; WGS, whole genome sequencing; MLST, multilocus sequence typing; MALDI-TOF MS, matrix-assisted laser desorption ionization time-of-flight mass spectrometry; qPCR, quantitative PCR.*

*Mixed infection CDT, cytolethal distending toxin.

*^a^Identified at species level.*

*^b^Result of the culture method.*

Russo et al. (2021) examined 225 cloacal swabs of yellow-legged gulls (*Larus michahellis*) in their study conducted in Italy and reported that 60 gulls (26.7%) were carriers of *Campylobacter*. Antilles et al. (2021) reported a *Campylobacter* carrier rate of 5.2% (93/1,785) in 1,785 cloacal swabs collected from gulls. In one study conducted in Iran, a relatively higher prevalence was reported in black-headed gulls (63.3%) and starlings (56.6%) compared to other bird species (Malekian et al., 2021). Another study from Turkey examined 183 cloacal swabs obtained from different species of wild birds and found a relatively higher prevalence (93%) in Eurasian coots (*Fulica atra*) compared to other birds (Kürekci et al., 2021). The study by Mencía-Gutiérrez et al. (2021) analyzed 689 bird of prey samples and reported a 7.5% prevalence. Another study from Poland investigated the *Campylobacter* prevalence in cloacal samples collected from black-headed gulls (718 adult and 318 chicks) and reported 4.87% (35/718) and 2.22% (7/318) prevalence in adults and chicks, respectively. This study found a non-significant difference among the age groups (adults and chicks) and birds’ habitats (urban and rural). However, a significant difference was found between breeding seasons (Indykiewicz et al., 2021). Broman et al. (2002), who compared the prevalences of *C. jejuni* isolated from juvenile and adult black-headed gulls, and Mencía-Gutiérrez et al. (2021), who assessed *Campylobacter* spp. prevalence difference among different age groups of birds of prey, also reported non-significant differences.

The prevalence variation among wild birds may be due to different factors such as location, season, wild bird species, sample type, the method used, ecological factors, and the health status of birds (Mohan et al., 2013; Cody et al., 2015; Mencía-Gutiérrez et al., 2021). For example, the distribution of *Campylobacter* may differ among wild bird species depending on ecological factors like their feeding habits and pattern of migration (Waldenström et al., 2002; Hald et al., 2016; Gargiulo et al., 2018). To support this argument, researchers have proved that wild birds that eat animal-origin food or forage on the ground near animal farms have a higher risk of acquiring *Campylobacter* than those foraging far away from the animal’s farm or hunt in the air (Hald et al., 2016). The differences between diurnal and nocturnal birds have also been assessed in several studies (Waldenström et al., 2002; Krawiec et al., 2017; Gargiulo et al., 2018). Gargiulo et al. (2018) compared diurnal and nocturnal birds and reported a statistically significant difference in the prevalence of *Campylobacter* species among diurnal (39.1%) and nocturnal (18.6%) birds. In contrast, the study conducted in Poland (Krawiec et al., 2017), in Sweden (Waldenström et al., 2002), and in Spain (Mencía-Gutiérrez et al., 2021) reported a higher prevalence in nocturnal birds. The study from New Zealand, which reported a significantly higher *Campylobacter* spp. prevalence in starlings (46%) than ducks (30%), documented a relatively higher prevalence during the spring and winter months than summer (Mohan et al., 2013). In agreement with this, Mencía-Gutiérrez et al. (2021) reported a significantly higher prevalence in samples they collected during the spring season. In contrast, Hald et al. (2016) reported a significantly higher prevalence during the summer season than the winter season.

## Drug Resistance Status

Antibiotic resistance, one of the growing global public health concerns (World Health Organization [WHO], 2020), has also been reported in *Campylobacter* isolates of wild bird origin (Table 1). Currently, several researchers have reported *Campylobacter* sp. resistance to different antibiotics (especially tetracycline and fluoroquinolones) (Wei et al., 2015; Jurado-Tarifa et al., 2016; Indykiewicz et al., 2021; Mencía-Gutiérrez et al., 2021; Russo et al., 2021). Multidrug resistance (MDR) *Campylobacter* isolates have been documented frequently (Wei et al., 2015; Du et al., 2019). Du et al. (2019) found 33.3% MDR in *Campylobacter* isolated from wild birds, with varying degrees of resistance to antibiotics like streptomycin, tetracycline, gentamicin, and clindamycin at rates of 36.84, 29.82, 29.82, and 28.07%, respectively. Antilles et al. (2021) also found 16.1% resistance to tetracycline in *Campylobacter* isolates isolated from gulls. In another study by Marotta et al. (2019), relatively higher resistance to tetracycline (19.40%) was recorded, with ciprofloxacin, nalidixic acid, and streptomycin resistance rates being 13.43, 10.45, and 10.45%, respectively.

A recent study by Mencía-Gutiérrez et al. (2021) reported resistance to nalidixic acid, ciprofloxacin, tetracycline, and streptomycin at the rates of 68.9, 68.9, 55.6, and 6.7%, respectively. However, a promising susceptibility to azithromycin (97.62%) and erythromycin (95.24%) was detected in a study conducted in Poland, in which only 50% resistance to tetracycline and 47.62% resistance to ciprofloxacin was reported (Indykiewicz et al., 2021). Similarly, the study from Lithuania reported 87.1% resistance to ciprofloxacin (Aksomaitiene et al., 2019). Another study from Italy reported resistance to tetracycline (12.5%), nalidixic acid (10%), ciprofloxacin (10%), streptomycin (6.7%), and erythromycin (4.2%) (Marotta et al., 2020). To sum up, the antibiotic resistance pattern of *Campylobacter* in wild birds seems under-investigated, and thus, further studies are warranted. However, as stated above, visible resistance to some antibiotics like tetracycline and fluoroquinolones is increasingly being reported.

## Virulence and Pathogenicity

Even though the pathogenesis of *Campylobacter* infection is not fully elucidated, several mechanisms are postulated to be involved (Asuming-Bediako et al., 2019), and virulence factors such as adhesion, bacterial invasion, and production of toxin are believed to have a role in its pathogenesis in humans (Kreling et al., 2020). Different virulence genes such as cytolethal distending toxin (CDT) genes, *flaA*, *flaB*, *ciaB*, and *cadF* have been documented in studies conducted with the aim of understanding the virulence of *Campylobacter* spp. isolated from the wild birds (Shyaka et al., 2015; Du et al., 2019). The genes encoding CDT (for example, *cdtA*, *cdtB*, and *cdtC*), the only toxin known to be produced by *Campylobacter* (Kreling et al., 2020), are frequently reported in wild birds (Weis et al., 2014; Shyaka et al., 2015). This toxin has DNAse activity that causes DNA damage (Kreling et al., 2020). As in other bacteria, adhesion to host epithelial cells is known to have a significant role in the pathogenesis of *Campylobacter*. However, unlike other bacteria (e.g., *E. coli* and *Salmonella*), fimbria does not mediate adhesion in the case of *Campylobacter* (Rubinchik et al., 2012), and the best-known adhesins in this bacterium are *Campylobacter* adhesion protein fibronectin (CadF) (Bolton, 2015; Kreling et al., 2020). This gene has also been reported in various wild bird species from countries like China (Du et al., 2019), Poland (Krawiec et al., 2017), and Japan (Shyaka et al., 2015). As discussed above, wild birds are considered to act as carriers, with the exception of general signs reported in some species (Taff and Townsend, 2017) that warrant further study, and information is scarce regarding clinical signs, pathogenicity, and pathology of *Campylobacter* infection in wild birds.

## Public and Animal Health Significance

Given the public health significance of *Campylobacter*, several studies that target comparing the genetic similarity of *Campylobacter* strains obtained from wild birds with strains circulating among poultry, humans, and other animals have been conducted (Broman et al., 2002, 2004; Waldenström et al., 2007; Colles et al., 2008, 2011; Griekspoor et al., 2013; Wei et al., 2019; Marotta et al., 2020; Zbrun et al., 2021). The results of some of these studies show high levels of host-specific strains (Broman et al., 2002, 2004; Waldenström et al., 2007; Messens et al., 2009; Griekspoor et al., 2013; Marotta et al., 2020). In contrast, some also report that *Campylobacter* spp. isolated from wild birds share similarities [e.g., sequence types (ST)] with those isolated from humans (French et al., 2009; Wei et al., 2019) and domestic animals (Sippy et al., 2012; Zbrun et al., 2021) and thus may serve as sources of drug-resistant potentially important pathogens even for humans (Cody et al., 2015; Mencía-Gutiérrez et al., 2021).

A molecular study by Colles et al. (2008) compared *Campylobacter* strains obtained from the wild bird (geese) with starlings and poultry populations using MLST and reported a high host specificity of *C. jejuni* genotypes obtained from wild geese. Broman et al. (2002) also investigated the genetic similarities between broiler (36 isolates), black-headed gull (*Larus ridibundus*) (76 isolates), and human (56 isolates) *C. jejuni* isolates in the same geographical region using PFGE. Their results showed a higher similarity profile between isolates obtained from humans and broiler when compared to isolates of humans and wild bird origin. However, they also emphasized that they found the same macrorestriction profile in 2 gull isolates and 1 human isolate. The result of another study conducted in Switzerland that compared the genetic similarity of *C. jejuni* isolates from migratory birds (89 isolates) and humans (47 isolates) showed that most of the strains from the migratory bird isolates were not related to the human strains, except the starling and blackbird strains, which showed similarity to some human strains (Broman et al., 2004).

The results of a large study covering 2,084 wild birds in the United Kingdom stated that the transmission pathway of *Campylobacter* is predominantly from farm animals to wild birds. In this study, 36 *C. jejuni* isolates were characterized by MLST, and the results showed that wild birds harbor both farm-related and unique *C. jejuni* strains. Nonetheless, the study did not witness wild bird-specific *C. jejuni* strains in farm animals (Hughes et al., 2009). Messens et al. (2009) also characterized *C. jejuni* obtained from wild birds and broilers and reported that the wild bird origin *C. jejuni* strains are different from broilers.

Unlike the above studies, a study conducted in South Korea has performed the genotypic analysis of *Campylobacter* species obtained from wild birds using MLST and reported ST similarity among humans and wild wilds (11 *C. jejuni* ST and 2 *C. coli* STs shown to be the same to those of human origin). The results of this study highlighted as *Campylobacter* isolated from wild birds are associated with domestic animal and environmental strains (Wei et al., 2019). A recent study by Zbrun et al. (2021) reported a genotypic similarity between *Campylobacter* isolated from broilers and wild birds, highlighting the possible role of wild birds in sustaining the epidemiology of this pathogen on farms. The same conclusion was made by Hald et al. (2016).

It has also been shown by Sippy et al. (2012) that wild birds carry *Campylobacter* isolates that share similarities with the *Campylobacter* strain known to be pathogenic for livestock. Similarly, in another study conducted in China, phylogenetic analysis of *C. jejuni* strains in different species of wild bird was performed, and it was determined that wild birds share the same ST with human-origin *C. jejuni*, indicating that this bacterium may be transmissible between different species (Du et al., 2019).

In a study conducted in Alaska, United States, *C. jejuni* isolates obtained from sick humans, environment, and wild birds during an outbreak of human campylobacteriosis in association with consumption of raw peas showed an indistinguishable PFGE, and the outbreak was linked with contamination from wild bird feces (Gardner et al., 2011). The outbreak of *C. jejuni* infection in children has also been linked to drinking “milk from bottles with bird-pecked tops” (Riordan et al., 1993). In another study from the United Kingdom, researchers investigated the role of wild birds as the source of human *Campylobacter* infection for nearly 10 years and found that wild birds accounted for 476 (2.1%) to 543 (3.5%) human cases per year (Cody et al., 2015).

As French et al. (2009) noted, the likely route of bird-to-human transmission can be equipment or surface contamination with wild birds’ fecal material (like in parks and children’s playgrounds). In this case, young children are more likely to be at risk because of frequent hand–mouth contact, which may expose them to swallowing infective material (French et al., 2009). In addition, wild birds are shown to be one of the leading sources of surface water contamination with *Campylobacter* spp. In a study conducted in the Netherlands that linked more than 90% of recreational water-origin *Campylobacter* isolates to wild birds, the risk of *Campylobacter* transmission by swimming in recreational water areas was emphasized (Mulder et al., 2020). In another study conducted in Canada that compared the similarity between *Campylobacter* strains (*C. lari*) isolated from river water and waterfowl, 100% homology was reported, and the likely risk of surface water contamination due to waterfowl was highlighted (Van Dyke et al., 2010). The study from Finland pointed out that swimming in natural water is independently related to sporadic campylobacteriosis (Schönberg-Norio et al., 2004). A similar finding was documented in a recent study by Mughini-Gras et al. (2021), who indicated that open-water swimming areas are a risk factor for human *Campylobacter* infections. Outbreaks of human campylobacteriosis that occurred in Norway in 1994 and 1995 were also suspected to be associated with drinking water contaminated with pink-footed geese feces (Varslot et al., 1996).

In summary, what we understand from research done so far seems controversial and inadequate to draw a complete conclusion about the risk of interspecies transmission of *Campylobacter* and warrants further comprehensive epidemiological investigations. As described in a framework proposed by Smith et al. (2020), criteria such as bacterial shedding pattern and bacterial survival in the environment need to be elucidated to better understand the possibility of transmission from wild birds to other hosts. Nevertheless, the detection of *Campylobacter* in wild birds is not neglectable from a public and animal health point of view. The main reason for this can be the isolation of drug-resistant *Campylobacter* species from various wild birds (Wei et al., 2015; Aksomaitiene et al., 2019; Du et al., 2019; Mencía-Gutiérrez et al., 2021). Furthermore, the results of the source attribution studies discussed above deserve public health attention. Therefore, despite the existing controversiality and the necessity of future studies, the detection of this pathogen in wild birds demonstrates their reservoir potential and the transmission of antibiotic-resistant pathogenic *Campylobacter* to domestic animals, and they may also play a role in *Campylobacter* transmission to humans by causing environmental contamination that may threaten public health (Gardner et al., 2011; Sippy et al., 2012; Du et al., 2019; Wei et al., 2019). To break the transmission chain, possible prevention and control interventions should target each transmission stage, and multi-sectoral collaborative epidemiological studies should be employed to monitor potential reservoirs using modern molecular techniques continuously.

## Conclusion and Future Perspectives

Currently, various wild bird species have been proven to be significant natural reservoirs of thermophilic *Campylobacter* species. In particular, *C. jejuni*, one of the major causes of foodborne infections worldwide, is the most frequently isolated species. Despite their reservoir role, the wild bird’s ability to transmit this pathogen to another host is not fully elucidated yet. Some studies have found overlapping strains among human, domestic animal, and wild bird isolates, while others have found significant differences in *Campylobacter* population structure among these hosts. In addition to pathogen detection, drug-resistant *Campylobacter* isolates, particularly resistance to tetracycline and fluoroquinolones, are documented. Source attribution studies have also linked human cases of *Campylobacter* infections to wild birds. Therefore, the role of wild birds in the epidemiology of *Campylobacter* should not be undermined. The currently available literature has focused on bacterial detection and, to some extent, antimicrobial resistance and comparative analysis of pathogen population structure in animals and humans. However, in order to determine disease status in wild birds and the precise role of wild birds in domestic animals and human health, detail-oriented molecular epidemiological studies characterizing the genetic relatedness of isolates from the respective species and environment through one health approach are warranted. In addition, determining bacterial survival in the environment, infective dose, and pathogen shading patterns in various bird species may play an essential role in clarifying the possibility of interspecies transmission. The study focusing on the clinical patterns of *Campylobacter* in infected wild birds also deserves attention from a conservation perspective.

## Author Contributions

NA: conceptualization, collecting the available data, and writing. TG: conceptualization and writing. Both authors have read and approved final version for publication.

## Conflict of Interest

The authors declare that the research was conducted in the absence of any commercial or financial relationships that could be construed as a potential conflict of interest.

## Publisher’s Note

All claims expressed in this article are solely those of the authors and do not necessarily represent those of their affiliated organizations, or those of the publisher, the editors and the reviewers. Any product that may be evaluated in this article, or claim that may be made by its manufacturer, is not guaranteed or endorsed by the publisher.
